# Multi-Omics Analysis Reveals the Negative Effects of High-Concentrate Diets on the Colonic Epithelium of Dumont Lambs

**DOI:** 10.3390/ani15050749

**Published:** 2025-03-05

**Authors:** Shufang Li, Hairong Wang, Boyang Li, Henan Lu, Jianxin Zhao, Aiwu Gao, Yawen An, Jinli Yang, Tian Ma

**Affiliations:** 1 Animal Nutrition and Feed Science, Inner Mongolia Agricultural University, Hohhot 010018, China; lishufang0325@163.com (S.L.); 13994489801@163.com (B.L.); lhena1117@163.com (H.L.); zhaojianxin226@sina.com (J.Z.); angjinli207@aliyun.com (J.Y.); matian226@sina.com (T.M.); 2 Food Science, Inner Mongolia Agricultural University, Hohhot 010018, China; nmndgaw@126.com; 3 Veterinary Research Institute, Inner Mongolia Academy of Agricultural & Animal Husbandry Sciences, Hohhot 010018, China; 18648041184@163.com

**Keywords:** microorganism, metabolomics, high-concentrate diets, colon, inflammation, Dumont lambs

## Abstract

Feeding HC diets has been found to induce metabolic dysregulation in the colon. However, the mechanisms by which changes in colonic flora and metabolites damage the colonic epithelium are poorly studied. Therefore, the present experiment used a multi-omics technique to investigate the mechanism of colonic injury induced by high-concentrate diets in lambs. Twelve male Dumont lambs were randomly split into two groups: a low-concentrate diet (LC = concentrate/forage = 30:70) group and a high-concentrate diet (HC = concentrate/forage = 70:30) group. The results showed that the HC group presented significantly increased lipopolysaccharide (LPS) concentrations in the colonic epithelium and significantly decreased serum total cholesterol (TC), superoxide dismutase (SOD), and glutathione peroxidase (GSH-Px) levels (*p* < 0.05), which led to cavities and inflammatory cell infiltration in the colonic epithelium. The HC group had significantly lower pH and less VFAs in colon contents, as well as a significantly increased abundance of bacteria of the genera *[Eubacterium]_coprostanoligenes_group*, *Rikenellaceae_RC9_gut_group*, *Treponema*, *Clostridia_UCG-014*, *Alistipes*, *Ruminococcus*, *Christensenellaceae_R-7_group*, *UCG-002*, *Bacteroidales_RF16_group* and *Lachnospiraceae_AC2044_group* compared to the LC diet group. These microorganisms significantly increased the level of metabolites of cholic acid, chenodeoxycholic acid, LysoPA (P-16:0/0:0), methapyrilene, and fusaric acid. A transcriptome analysis showed that cytokine–cytokine receptor interaction, glutathione metabolism, and the peroxisome signaling pathway were downregulated in the colon epithelium of the lambs fed the HC diet. Therefore, the HC diet caused epithelial inflammation and oxidative damage by affecting the interaction between the microbial flora of the colon and metabolites and the host epithelium, which eventually disrupted colon homeostasis and had a negative impact on sheep health.

## Simple Summary

There are limited systematic studies on the effects of high-concentrate (HC) diets on the colonic health of ruminants. Our study found that HC diets induced a decrease in colonic pH and the accumulation of volatile fatty acids (VFAs), leading to dysbiosis of the microbiota characterized by a reduced abundance of cellulolytic bacteria and an increased abundance of starch-degrading bacteria and opportunistic pathogens. Abnormal metabolites associated with colonic epithelial injury were significantly enriched, and a transcriptomic analysis revealed the inhibition of glutathione metabolism and peroxisome-based antioxidant defense systems, accompanied by an aberrant activation of cytokine receptor interaction pathways. Colonic epithelial integrity was compromised with inflammatory infiltration in the HC group, and serum antioxidant enzyme activity was reduced. These findings suggest that HC diets disrupt colonic microbiome–metabolome homeostasis, impair mucosal barrier function, and induce oxidative stress, ultimately threatening the colonic stability and health of ruminants.

## 1. Introduction

Feeding HC diets to satisfy the increased nutritional demands of high-production ruminants is a common practice in livestock management. However, this dietary composition alteration often increases the incidence of nutritional metabolic disorders [1]. Digestion in the hindgut is essential for ruminants; approximately 17% of digestible cellulose is metabolized in the cecum and 13% is metabolized in the colon [2], resulting in the production of around 12% of VFAs [3] and contributing about 8% to the metabolic energy requirements of sheep [4]. When ruminants consume a high proportion of grain feed and a small amount of forage, the VFA and lactic acid concentrations in the rumen increase and the pH decreases, inducing subacute ruminal acidosis (SARA), which causes metabolic disorders of rumen microorganisms and reduces the absorption and barrier ability of rumen epithelial cells [1]. At the same time, the amount of rumen bypass starch entering the hindgut increases [5,6,7]. Due to the absence of saliva and protozoan buffering in the hindgut, coupled with the presence of a mucus layer in the intestinal lumen, which leads to a decrease in pH, VFA absorption is not promoted, causing hindgut acidosis [8,9]. Furthermore, the hindgut epithelium is a monolayer columnar epithelium, which renders the permeability and integrity of the hindgut mucosa more susceptible to compromise [1].

The cecum and colon are colonized by a rich and complex microbiome, dominated by bacteria (more than 95%), including archaea [10]. During digestion and metabolism, intestinal microorganisms communicate with host cells via the production of metabolites and signaling molecules, which affect the metabolism and immunity of the host [11]. Intestinal microorganisms are influenced by many factors, such as diet [12,13,14], feeding method [15], season [16] and genetics [17]. HC diets have been shown to significantly reduce microbial diversity and richness in the ruminant hindgut [12], accompanied by a marked decline in the relative abundance of fibrinolytic bacteria [18] and an increase in amylolytic and pathogenic bacteria, which ultimately affects the intestinal health of the host [19,20]. However, most current studies examining the effects of HC diets on ruminants have primarily focused on the rumen, with less information on metabolic dysregulation in the colon and even less still on the interactions of changes in colon flora and metabolites induced by HC diets with the host epithelium. Therefore, this experiment investigated the interactions of colonic microorganisms and their metabolites with the host on an HC diet.

## 2. Materials and Methods

### 2.1. Animal Feeding and Experimental Design

An experiment was conducted at Hailutu Farming Base (40°40′30″ N, 111°21′34″ E) with 12 male Dumont lambs (who had an average weight of 26.37 ± 2.29 kg and were 3 months old). The lambs, sourced from Inner Mongolia Sino Breeding Sheep Technology Co., Ltd (Ulanqab, Inner Mongolia, China). were randomly assigned to two groups. The lambs were housed in individual metabolic cages (1.5 m × 1 m × 1 m) with ad libitum access to water and fed twice daily at 08:00 and 18:00. The control group received a low-concentrate diet (LC = concentrate/forage = 30:70), while the experimental group received an HC diet (HC = concentrate/forage = 70:30). The pre-feeding period lasted 15 days, followed by a 60-day experimental period. Diet composition and nutritional details are provided in Table A1.

### 2.2. Sample Collection

After the experiment, blood was collected from the jugular vein of the lambs immediately post-mortem (the LC group’s average weight was 36.70 ± 1.20 kg, and the HC group’s average weight was 41.79 ± 3.89 kg). Lambs were exsanguinated according to the Interim Regulations on the Management of Livestock and Poultry Slaughtering in Hohhot issued by the People’s Government of Hohhot, China. Colonic contents were collected for pH measurement. Furthermore, the colon tissue was washed with PBS post-mortem. Approximately 1 cm of the intestinal segment of the colon was collected and fixed using a 4% paraformaldehyde solution for a histological analysis. Colon contents were collected with a sterile medicine spoon and placed into a freezing tube for quick freezing with liquid nitrogen. Colonic epithelial tissues were rinsed with pre-cooled phosphate-buffered saline (PBS) and then epithelium was separated from underlying layers by scraping using a coverslip, and the cells were frozen in a tube containing 0.5 mL RNase inhibitor (BeyotimeBiotechnology, Shanghai, China) and subsequently stored at −80 °C.

### 2.3. Histological Analysis of Colon Tissue

The fixed colon tissues were subjected to paraffin sectioning and hematoxylin–eosin (HE) staining according to conventional methods. Colon morphology was observed under a microscope (Nikon, Minato-ku, Tokyo Metropolis, Japan).

### 2.4. Measurement of Colonic Epithelial LPS Content and Serum Parameters

Serum total protein (TP), albumin (ALB), globulin (GLB), glucose (GLU), total cholesterol (TC), and triglyceride (TG) levels were measured using a biochemical analyzer, with kits supplied by Lepu Diagnostic Technology Co., Ltd., (Beijing, China). The kits for serum immunoglobulin A (IgA), immunoglobulin M (IgM), immunoglobulin G (IgG), serum smyloid A (SAA), interleukin-1β (IL-1β), tumor necrosis factor–α (TNF-α), interleukin–6 (IL-6), superoxide dismutase (SOD), glutathione peroxidase (GSH-Px), and colon LPS concentrations were provided by Wuhan Gene Biotechnology Co., Ltd., (Wuhan, Hubei, China).

### 2.5. Quantitative Real-Time PCR Analysis

Total colonic RNA was extracted using Trizol (TaKaRa Bio, Kyoto, Kyoto Prefecture, Japan) and reverse-transcribed into cDNA after confirming the concentration and purity using a Nanodrop 2000 (Thermo Scientific, Waltham, MA, USA). The primer sequences are listed in Table A2. qRT-PCR was performed, with β-actin and glyceraldehyde-3-phosphate dehydrogenase (GAPDH) as the internal references. The amplification conditions were 95 °C for 30 s (pre-denaturation), followed by 40 cycles of 95 °C for 5 s and 60 °C for 20 s. Gene expression was calculated using the 2^−ΔΔCt^ method.

### 2.6. Measurement of VFA Concentration

The VFA concentrations in the colon were analyzed using gas chromatography, following the method of Tao et al. [7]. First, 2.0 g of the colon content was mixed with 2 mL of distilled water, thawed at 4 °C, and vortexed. After centrifugation at 10,000× *g* for 20 min at 4 °C, 1.5 mL of the supernatant was mixed with 200 μL of 2-EB, vortexed, and filtered through a 0.22 μm membrane into a sample vial. An analysis was performed using a Shimadzu GC-2014C gas chromatograph (Shimadzu, Kyoto, Kyoto Prefecture, Japan), equipped with a DB-FFAP capillary column (30 m × 0.50 μm × 0.250 mm). The chromatographic conditions were as follows: The injection port and detector temperatures were set to 220 °C and 250 °C, respectively. The column temperature was maintained at 60 °C for 1 min and then increased to 115 °C at 5 °C/min, followed by a rise to 180 °C at 15 °C/min. The VFA concentrations were determined by measuring the peak areas and comparing them to a standard curve.

### 2.7. Colon Microbial Diversity Analysis

Total DNA from the colonic content was extracted using a Magen kit (Guangzhou Magen Biotechnology Co., Ltd., Guangzhou, Guangdong, China). PCR amplification was performed using the primers 343 f (5′-tagggragcag-3′) and 798 r (5′-agggtattctactt-3′) after confirming the concentration and purity. The sequencing was performed by Shanghai OE Biotechnology Co., Ltd. on an Illumina MiSeq platform (Illumina, CA, USA). Raw fastq data were processed with Cutadapt for adapter removal, followed by DADA2 in QIIME2 for quality filtering, denoising, read merging, and chimeric sequence removal [21]. Representative sequences and an ASV abundance table were generated. Alpha diversity (Chao, Shannon, Simpson, and observed indices) indices were calculated using QIIME2’s core-diversity plugin [22]. Beta diversity was calculated using the principal coordinate analysis (PCoA) approach. Important bacterial taxa among the groups were identified using LEfSe (LDA ≥ 4). Bioinformatics analyses were performed on the OE Cloud platform (Available online: https://cloud.oebiotech.com/#/home).

### 2.8. Metabolomics Analysis of Colon Content

Furthermore, 30 mg of colon contents was mixed with 400 ul methanol aqueous solution (1:4, *v*/*v*), frozen at −20°C for 2 h, then ground for 2 min. Ultrasonic extraction was performed in an ice–water bath for 10 min, followed by centrifugation at 12,000× *g* for 10 min at 4 °C to collect the supernatant. The collected supernatant was analyzed by Shanghai OE Biotechnology Co., Ltd (Shanghai, China). using LC-MS/MS. The original LC-MS data were processed with Progenesis QI V2.3 software (Nonlinear Dynamics, Newcastle, UK) for baseline filtering, peak identification, integration, retention time correction, peak alignment, and normalization. A qualitative analysis was performed using the Human Metabolome Database (HMDB), Lipidmaps (V2.3), Metlin, EMDB, PMDB, and a self-built database to further obtain metabolite information. Metabolites were analyzed using a KEGG pathway enrichment analysis and MetaboAnalyst 4.0 (Available online: http://www.metaboanalyst.ca/) to identify key biochemical and signaling pathways.

### 2.9. Colon Transcriptome Sequencing

Total RNA was isolated from the colon tissue using Trizol reagent (TaKaRa Bio, Kyoto, Kyoto Prefecture, Japan)), and RNA concentration and purity (A260/A280) were assessed with a Nanodrop 2000. The sequencing was performed on an Illumina NovaSeq 6000 platform (Illumina, San Diego, CA, USA) by Shanghai OE Biotechnology Co., Ltd. The raw fastq reads were processed using Fastp, and low-quality reads were removed to obtain clean reads. These clean reads were mapped to the reference sheep genome (Available online: https://www.ncbi.nlm.nih.gov/datasets/genome/?taxon=9940 (accessed on 6 April 2023) using HISAT2. The FPKM values of each gene were calculated, and read counts were generated using HTSeq-count. Differential gene expression was analyzed using DESeq, with *p* < 0.05 and FoldChange > 1.5 set as the thresholds for identifying significantly different genes between groups. The functional enrichment and signaling pathways of the differentially expressed genes were analyzed using the KEGG database. All bioinformatics analyses were conducted on the OE Cloud platform (Available online: https://cloud.oebiotech.com/#/home).

### 2.10. Statistical Analyses

Analyses of the data on serum biochemical, immune, and antioxidant indices, colon pH, and VFA content were conducted using the *t*-test based on SPSS 23 (IBM Corporation, Armonk, NY, USA). Violin plots were generated using GraphPad Prism (V 8.0.1, GraphPad, San Diego, CA, USA). *p* < 0.05 was considered significantly different, and *p* < 0.01 was considered highly significantly different.

For a microbial analysis, the Wilcoxon rank-sum test was employed to compare the relative abundances of the colon microbiota at the phylum and genus levels. FDR-adjusted *p* < 0.05 was considered significantly different. Spearman’s correlation coefficient was used to evaluate the relationship between the colon bacterial composition and VFAs, with significant correlations of *p* < 0.05 and R > 0.60.

OPLS-DA analyses were performed using the “ropls” package in R (Version 1.6.2). A two-tailed Student’s *t*-test was used to calculate the *p*-values for the differential metabolites between the LC and HC groups. Metabolites with VIP > 1 and *p* < 0.05 were considered significantly different. Spearman’s correlation coefficient was used to evaluate the relationship between the colon bacterial composition and differential metabolites, with significant correlations of *p* < 0.05 and R > 0.60.

## 3. Results

### 3.1. Effect of HC Diet on Colon Epithelial Lipopolysaccharide Content and Serum Parameters in Dumont Lambs

**Figure 1:**
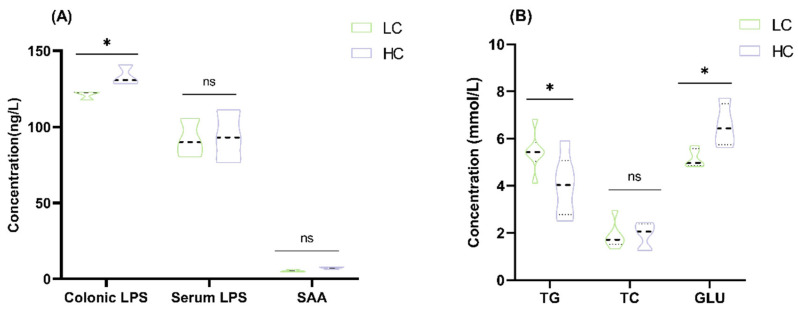

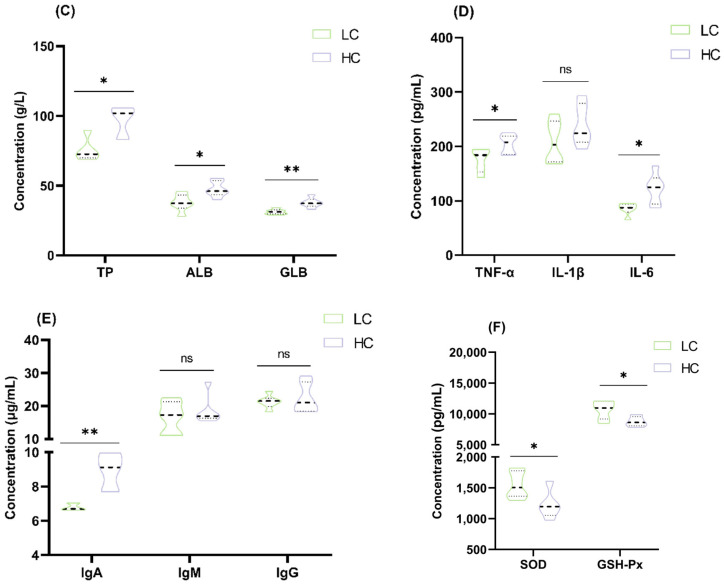
Effect of HC diet on colon epithelial lipopolysaccharide content and serum parameters in Dumont lambs. (**A**) The concentrations of colonic epithelial LPS, serum LPS, and SAA. (**B**) The concentrations of serum TG, TC, and GLU. (**C**) The concentrations of serum TP, ALB, and GLB. (**D**) The concentrations of serum TNF-α, IL-1β, and IL-6. (**E**) The concentrations of serum IgA, IgM, and IgG. (**F**) The concentrations of serum SOD and GSH-Px. LC and HC represent diets with concentrate/forage ratios of 30:70 and 70:30. Not significantly different (ns.) *p* > 0.05, * *p* < 0.05, ** *p* < 0.01.

Colonic epithelial LPS concentrations were significantly elevated in the HC group (*p* < 0.05; Figure 1A). GLU, TP, ALB, and GLB levels were significant higher in the HC group, while TG was lower compared to the LC group (*p* < 0.05 or *p* < 0.01; Figure 1B,C). Additionally, TNF-α, IL-6, and IgA concentrations increased, while serum SOD and GSH-Px levels decreased in the HC group (*p* < 0.05 or *p* < 0.01; Figure 1D–F).

### 3.2. Effects of HC Diet on Colonic Epithelium Morphology and Tight Junction mRNA Expression in Dumont Lambs

**Figure 2:**
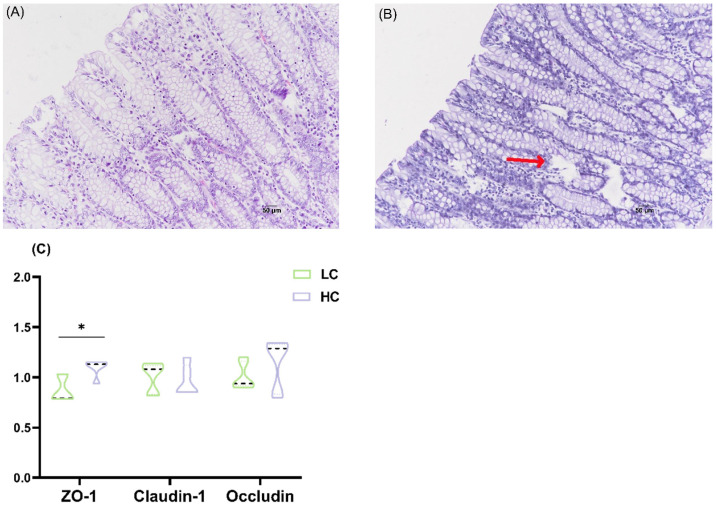
Effects of HC diet on the morphology of the colonic epithelium and tight junction mRNA expression in Dumont lambs. (**A**) LC group, cavities and inflammatory cell infiltration appeared. (**B**) HC group. (**C**) The expression of ZO-1, Cloudin-1, and Occludin mRNA. LC and HC represent diets with concentrate/forage ratios of 30:70 and 70:30. Significantly * *p* < 0.05.

We found that, in the HC group, the colonic epithelium showed cavities and inflammatory cell infiltration (Figure 2B). In the HC group, zo-1 mRNA expression increased significantly (*p* < 0.05; Figure 2C).

### 3.3. Effect of HC Diet on Colon Fermentation Parameters and Microbial Composition in Dumont Lambs

**Figure 3:**
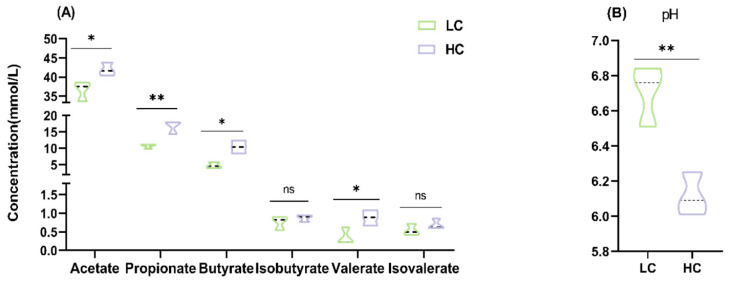
Effect of HC diet on colonic fermentation parameters of Dumont lambs (colon contents collected post-mortem). (**A**) VFAs in the colon content. (**B**) Colon pH. LC and HC represent diets with concentrate/forage ratios of 30:70 and 70:30. Not significantly different (ns.) *p* > 0.05, * *p* < 0.05, ** *p* < 0.01.

Compared to the LC group, the acetate, propionate, butyrate, and valerate concentrations were significantly higher (*p* < 0.05 or *p* < 0.01), while pH was significantly lower in the colonic content of the HC group (*p* < 0.01; Figure 3A,B).

**Figure 4:**
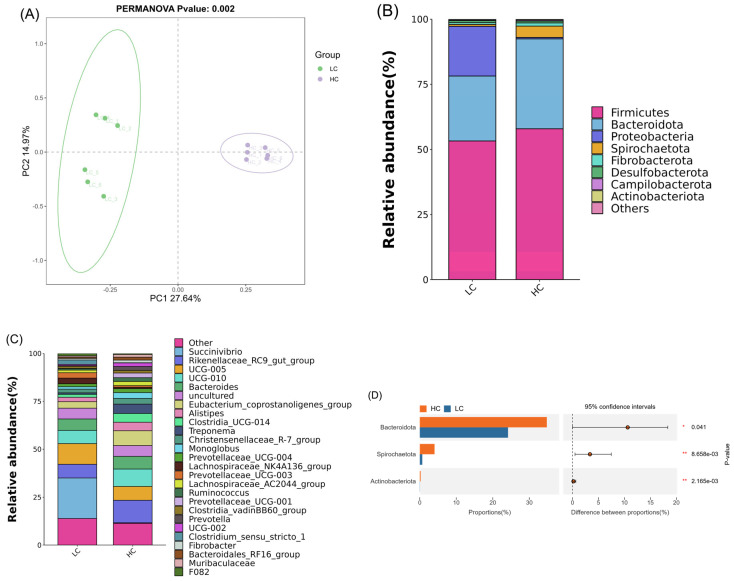

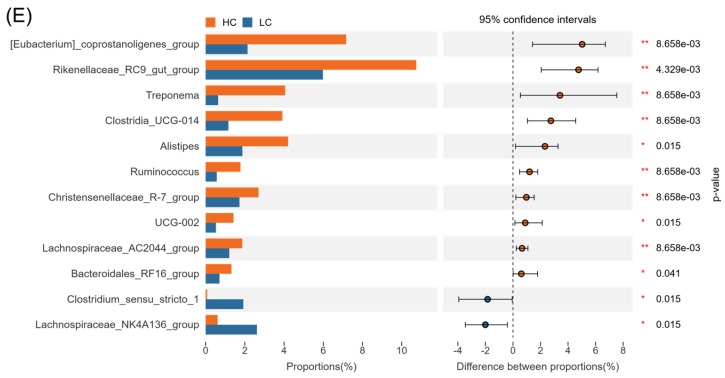
Effect of HC diet on microbial composition in colon of Dumont lambs. (**A**) PCoA plot. (**B**) Bacterial phylum with relative abundance ≥ 0.1%. (**C**) Bacterial genus with relative abundance ≥ 0.1%. (**D**) Comparison of bacterial phylum abundance with relative abundance ≥ 0.1% between LC and HC groups. Bacterial abundance at phylum level. (**E**) Comparison of bacterial genus abundance with relative abundance ≥ 1% between LC and HC groups. LC and HC represent diets with concentrate/forage ratios of 30:70 and 70:30. * *p* < 0.05, ** *p* < 0.01.

**Figure A1:**
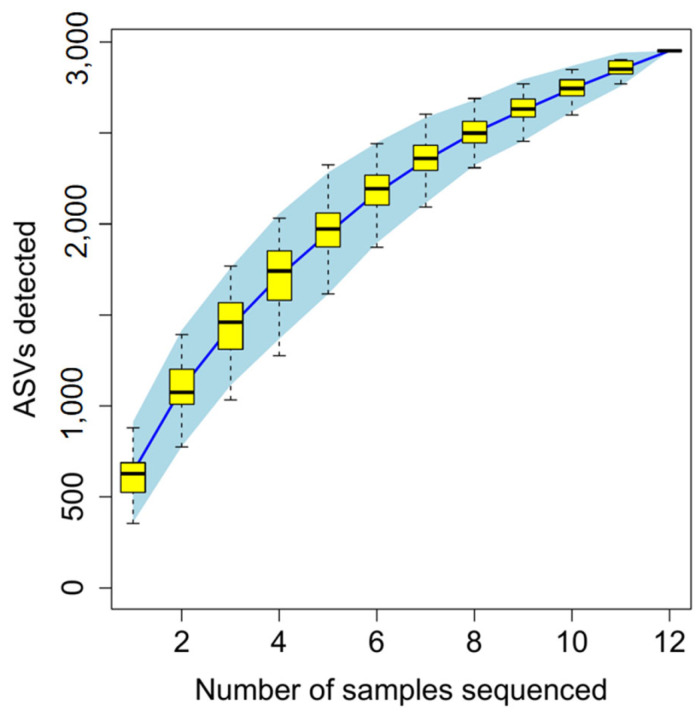
Species accumulation curve. The horizontal axis represents the sample size and the vertical axis represents the number of ASVs detected.

**Figure A2:**
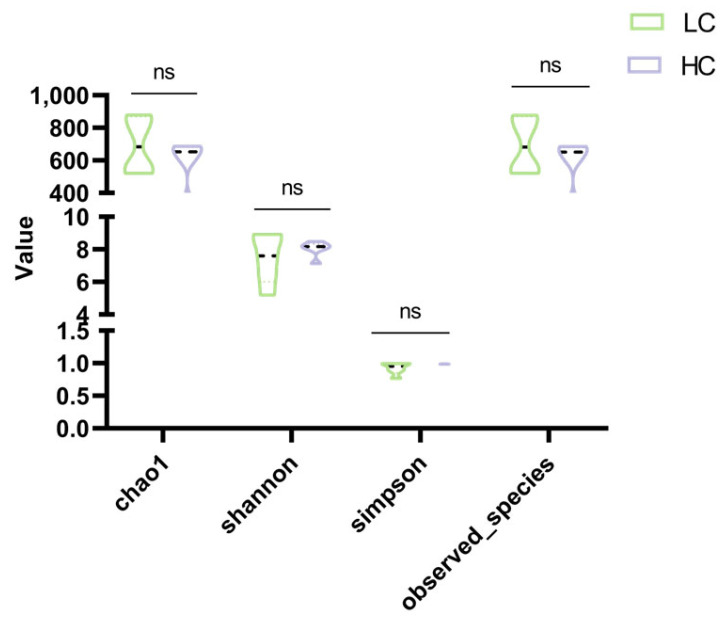
The comparison of α-diversity (Chao, Shannon, Simpson and observation index) of bacterial communities at ASVs level. LC and HC represent diets with concentrate/forage ratios of 30:70 and 70:30. Not significantly different (ns.) *p* > 0.05.

According to the 16S rRNA sequencing results, 956,037 raw reads and 845,953 high-quality sequences were obtained from the two groups of lamb colon contents, and 2952 amplicon sequence variants (ASVs) were generated following quality control. Sample species accumulation curves showed that the sampling was reasonable and sufficient (Figure A1). The effect of the high-concentration diet on colonic microbial diversity was assessed via α-diversity and β-diversity. The results showed no significant change in α-diversity (Figure A2), but significant microorganisms were separated between the two groups (Figure 4A).

At the phylum level, eight dominant phyla were identified (≥0.1% relative abundance in at least one group, Figure 4B). *Firmicutes* and *Bacteroidota* were the main phyla in the LC and HC groups, accounting for 53.2% and 24.9% in the LC group and 57.9% and 34.5% in the HC group, respectively. An abundance difference analysis at the phylum level showed that the relative abundances of *Bacteroidota* (*p* < 0.05), *Spirochaetota* (*p* < 0.01), and *Actinobacteriota* (*p* < 0.01) in the HC group were significantly higher than those in the LC group (Figure 4D). At the genus level, 26 dominant genera were identified (≥1% relative abundance in at least one group) (Figure 4C). An abundance difference analysis at the genus level showed that, in the HC group, the relative abundances of *[Eubacterium]_coprostanoligenes_group* (*p* < 0.01), *Rikenellaceae_RC9_gut_group* (*p* < 0.01), *Treponema* (*p* < 0.01), *Clostridia_UCG-014* (*p* < 0.01), *Alistipes* (*p* < 0.05), *Ruminococcus* (*p* < 0.01), *Christensenellaceae_R-7_group* (*p* < 0.01), *UCG-002* (*p* < 0.05), *Bacteroidales_RF16_group* (*p* < 0.05), and *Lachnospiraceae_AC2044_group* (*p* < 0.01) increased significantly, while the relative abundances of *Lachnospiraceae_NK4A136_group* (*p* < 0.05) and *Clostridium_sensu_stricto_1* (*p* < 0.05) decreased significantly (Figure 4E).

**Figure 5:**
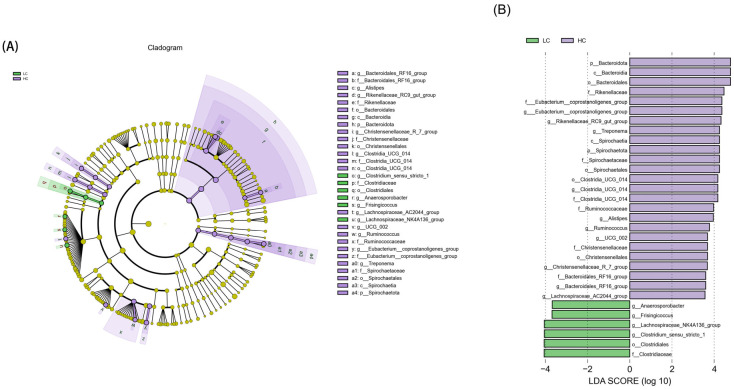
LEfSe analysis of microbial community in colon of lambs in LC and HC groups. (**A**) Cladogram of LDA scores. (**B**) Bar graph of the LDA value distribution.

An LEfSe analysis identified 31 biomarkers with significant differences between the LC and HC groups (LDA score > 3.5). The cladogram in Figure 5A clearly shows the distribution of differentially abundant taxa across different taxonomic levels. The purple branches represent the taxa enriched in the HC group, while the green branches represent those enriched in the control group. Figure 5B presents the LDA scores of the identified biomarkers.

**Figure 6:**
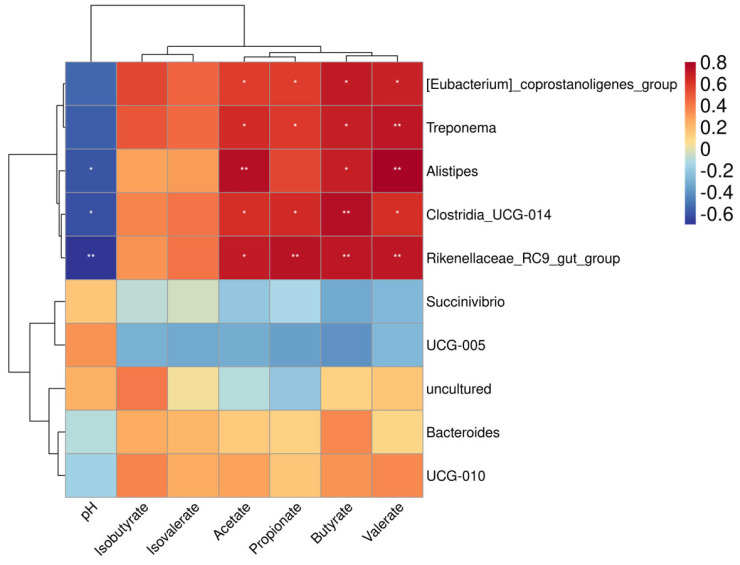
Correlation analysis between the relative abundances of colonic microbial genera and colonic fermentation parameters. Spearman’s correlation between VFAs and top 10 genera; * *p* < 0.05, ** *p* < 0.01, R > 0.60.

Spearman’s correlation was used to evaluate the relationship between bacterial abundance and fermentation parameters (Figure 6). The heat map revealed positive correlations between *[Eubacterium]_coprostanoligenes_group, Treponema, Clostridia_UCG-014,* and *Rikenellaceae_RC9_gut_group* and acetate, propionate, butyrate, and valerate (*p* < 0.05 or *p* < 0.01). *Alistipes* were positively correlated with acetate butyrate and valerate (*p* < 0.05 or *p* < 0.01). Additionally, *Alistipes, Clostridia_UCG-014,* and *Rikenellaceae_RC9_gut_group* showed negative correlations with pH (*p* < 0.05 or *p* < 0.01).

### 3.4. Effect of HC Diet on Metabolites in Colon Content of Dumont Lambs

**Figure 7:**
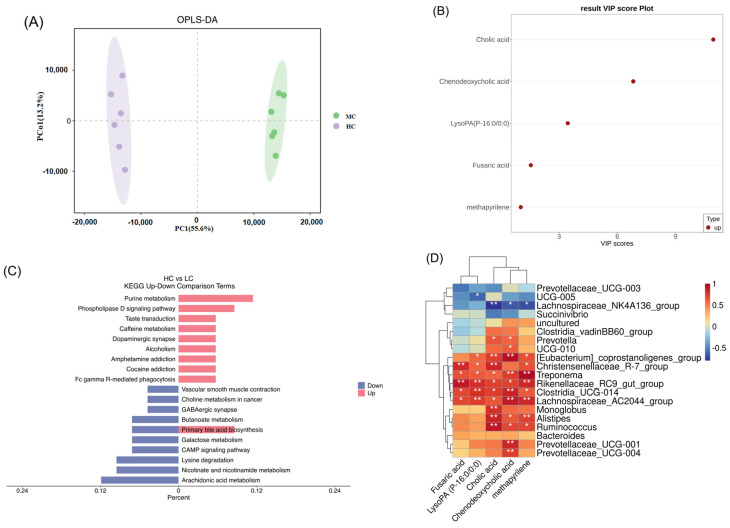
Effect of high-concentrate diet on metabolites in colon content of Dumont lambs. (**A**) OPLS-DA based on the metabolite matrix of colon content. (**B**) Differential metabolite VIP value of LC and HC groups. (**C**) KEGG enrichment analysis of differential metabolites of LC and HC groups, where red represents upregulated, and blue represents downregulated. (**D**) Correlation network analysis between metabolites and the top 20 bacterial genera showed positive correlations (red squares) and negative correlations (blue squares). A significant correlation was defined as R > 0.6, * *p* < 0.05, ** *p* < 0.01. LC and HC represent diets with concentrate/forage ratios of 30:70 and 70:30.

**Figure A3:**
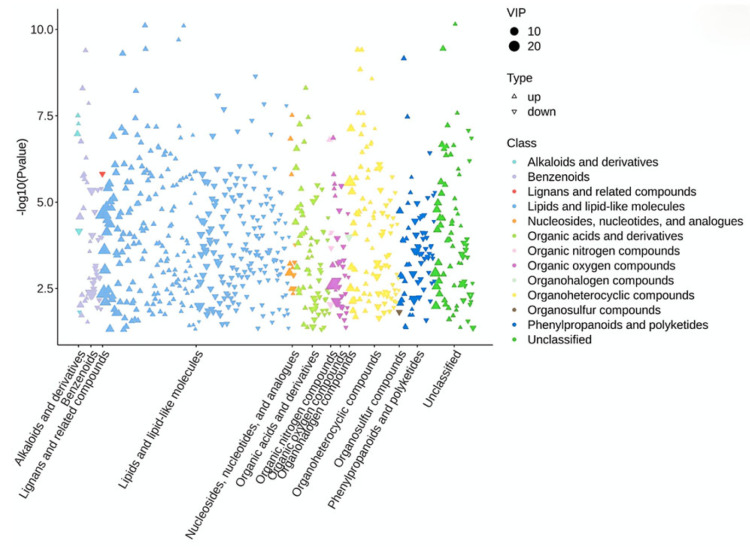
Classification of significantly different metabolites.

A non-targeted metabolomic analysis of the colon content was performed using an LC-MS system, and 1019 metabolites were detected. OPLS-DA revealed significant metabolite differences between the two groups (Figure 7A), identifying 779 differential metabolites with VIP > 1 and *p* < 0.05, primarily consisting of lipids, lipid-like molecules, and organoheterocyclic compounds (Figure A3). Among these, 396 metabolites were upregulated, and 383 were downregulated. Among the upregulated differential metabolites, we screened five differential metabolites that may have adverse effects on the colon: cholic acid, chenodeoxycholic acid, LysoPA (P-16:0/0:0), methapyrilene, and fusaric acid (Figure 7B).

The KEGG pathway enrichment analysis showed that the differential metabolites were mainly enriched in purine metabolism, primary bile acid biosynthesis, the phospholipase D signaling pathway, arachidonic acid metabolism, nicotinate and nicotinamide metabolism, lysine degradation, and butanoate metabolism (Figure 7C). A correlation analysis revealed that *Ruminococcus* and *Alistipes* were significantly positively correlated with methapyrilene, cholic acid, and chenodeoxycholic acid. *Treponema*, *Rikenellaceae_RC9_gut_group*, *Clostridia_UCG-014,* and *Lachnospiraceae_AC2044_group* were significantly positively correlated with LysoPA (P-16:0/0:0), fusaric acid, methapyrilene, cholic acid, and chenodeoxycholic acid. *Lachnospiraceae_NK4A136_group* was significantly positively correlated with cholic acid, chenodeoxycholic acid, and methapyrilene. *UCG-005* was significantly negatively correlated with LysoPA (P-16:0/0/0:0) (Figure 7D).

### 3.5. Effect of HC Diet on the Transcription Profile of the Colon Epithelium

To further investigate the effects of colon microorganisms and metabolites on substance metabolism and the signal transduction of host epithelial cells, we sequenced the transcriptome of the colon epithelial samples. The sequencing results showed that 44.46 Gb of raw data was obtained from six colon epithelial samples, with a Q30 percentage > 95%.

**Figure 8:**
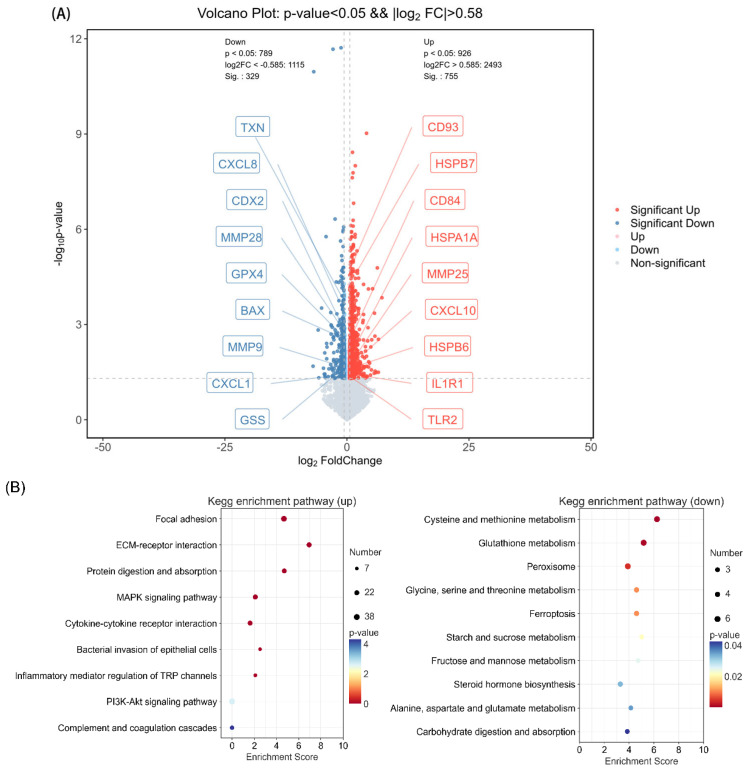
Effect of a high-concentrate diet on transcriptional profile of colon epithelium in Dumont lambs. (**A**) Volcano plots from RNA-seq analysis of the HC and LC groups show upregulated DEGs in red and downregulated DEGs in blue. (**B**) The KEGG enrichment analysis of DEGs of LC and HC groups. GPX4 = glutathione peroxidase 4; IL1R1 = interleukin 1 receptor type 1; TLR2 = Toll-like receptor 2; MMP25 = matrix metalloproteinase 25; CXCL10 = chemokine (C-X-C motif) ligand 10; MMP9 = matrix metalloproteinase 9; MMP28 = matrix metallopeptidase 28; HSPA1A = heat shock protein 1A; TXN = thioredoxin; CXCL8 = chemokine (C-X-C motif) ligand 8; HSPB6 = heat shock protein beta 6; CDX2 = caudal-type homeobox 2; HSPB7 = heat shock protein beta 7; CD93 = cluster of differentiation 93; CXCL1 = chemokine (C-X-C motif) ligand 1; CD84 = cluster of differentiation 84; GSS = glutathione synthetase; BAX = Bcl-2-associated X protein. LC and HC represent diets with concentrate/forage ratios of 30:70 and 70:30.

From the two groups, 1084 differentially expressed genes (DEGs) were identified, with 755 being upregulated and 329 being downregulated (Figure 8A). Among them, we screened out 18 DEGs related to immunity, antioxidation, and epithelial cell repair. The upregulated DEGs included CD93, HSPB7, CD84, HSPA1A, MMP25 Level, CXCL10, HSPB6, IL 1R1, and TLR2, while the downregulated DEGs comprised TXN, CXCL8, CDX2, MMP28, GPX4, Bax, MMP9, CXCL1, and GSS (Figure 8A). All DEGs were enriched in 286 KEGG pathways, among which 20 pathways were screened. Significantly enriched pathways with up-regulated genes included focal adhesion, cytokine–cytokine receptor interaction, bacterial invasion of epithelial cells, and inflammatory mediator regulation of TRP channels; the significantly downregulated pathways included cysteine and methionine metabolism, glutathione metabolism, and peroxisome (Figure 8B).

## 4. Discussion

The colon contains many microorganisms, which ferment carbohydrates, protein, and amino acids such as those from the rumen to form short-chain fatty acids, hydrogen sulfide, and other metabolites [23,24]. When ruminants eat a high-grain-concentrate diet and a small amount of forage, carbohydrates flow into the small intestine and hindgut [5,6,7]; carbohydrates in the presence of hindgut microorganisms produce VFAs, lactic acid, and metabolites that trigger inflammation and immune responses, affecting host intestinal health [25,26]. In this study, the negative effects of an HC diet on the colon epithelium were discussed by analyzing the bacterial community, the metabonomics of the colon content, and the transcriptome of the colon epithelium.

Normally, in the hingut are undigested polymers such as lignin and crystalline starch that is incompeletly digested in the rumenand small intestine. In addition host-secreted mucins and sed cells contribute to digestible substrates available in the colon. In addition, host-secreted mucins and sed cells contribute to digestible substrates available in the colon. When the dietary structure changes, such as when high-yielding animals consume a high proportion of grains and a small amount of forage, this results in more fermentable substrate flowing into the hindgut [7], which leads to the accumulation of organic acids, a decrease in pH, and even hindgut acidosis [8,27,28]. This study found that an HC diet increased the acetate, propionate, butyrate, and valerate concentrations in the colonic content and decreased the pH compared to an LC diet. These results are similar to those of Chen et al. [12], Lin et al. [14] and Tao et al. [19].

A decreased gut pH causes the death and lysis of acid-sensitive Gram-negative bacteria, leading to the release of large amounts of LPS. LPS can be recognized by immune cells or intestinal epithelial cells [29,30]; when the intestinal epithelium is damaged, LPS undergoes translocation into the bloodstream and activates NF-κB through the LPS/Toll-like receptor 4 (TLR4) signaling pathway, triggering local or systemic inflammation [31,32]. Previous studies found that LPS concentrations in portal vein serum and colon content significantly increased following an HC diet, along with an elevated expression of pro-inflammatory cytokines in the colon epithelium [12]. This indicates that feeding a high-concentrate diet causes colonic epithelial inflammation. This study found that the LPS concentration in the colon epithelium increased in the HC group, but serum LPS and SAA exhibited no significant changes. Although serum LPS did not significantly change in this study, our previous study found that high-concentrate diets significantly increased serum LBP, TNF-α, and IL-6 concentrations, suggesting a shift in LPS levels [33,34]. SAA is a marker of acute inflammation, and there was no significant change in SAA when lambs were fed with a high-concentrate diet for 60 days. In addition, we also found that the colon epithelium in the HC group showed cavities and severe cell damage. Interestingly, the HC diet was associated with an increased expression of zo-1mRNA in the colon epithelium. The expression of ZO-1 is related to tight junction permeability, and it is generally believed that its increase represents a decrease in permeability [35]. Therefore, the relationship between the damage to the colon epithelium and the increase in zo-1 expression needs to be verified by quantitation of changes in the abundance of the zo-1 protein.

Intestinal microorganisms play a vital role in nutrient degradation, utilization, and immune regulation [16], and they are influenced by various factors such as diet [13], feeding mode [15], season [36,37] and heredity [17]. Intestinal microorganisms change in microbial flora populations and metabolism with diet composition [38]. Studies indicate that an HC diet significantly alters ruminants’ hindgut microbiota, which are usually characterized by changes in the dominant flora at the phylum level and a reduced diversity of bacterial communities [12,14,18]. This study found that the α-diversity (examined via the Chao, Shannon, Simpson, and observation indices) did not significantly change, but a PCoA and Adonis analysis showed that the HC diet altered the microbiological composition of the colon, indicating that the diversity of colonic flora was closely related to the diet structure. At the phylum level, the HC group exhibited a significantly higher relative abundance of *Bacteroidota*, *Spirochaetota*, and *Actinobacteriota*. *Bacteroides* secretes many enzymes related to carbohydrate fermentation [39], which can utilize a variety of carbohydrates from food and the host; acetate, propionate and butyrate being the primary metabolites [40,41]. This is consistent with the increase in the proportion of acetate, propionate, and butyrate in the HC group colon content. This study’s results align with those of Chen et al. [12], who found that an HC diet increased the abundance of *Spirochaetota*. Although *Actinobacteriota* account for a relatively small proportion, they may be crucial for intestinal balance [42].

Among the dominant genus with relative abundance ≥ 1%, HC significantly enhanced the abundance of Rikenellaceae_RC9_gut_group, Alistipes, Treponema, [Eubacterium]_coprostanoligenes_group, Ruminococcus, Christensenellaceae_R-7_group, UCG-002, Lachnospiraceae_AC2044_group, Bacteroidales_RF16_group and Clostridia_UCG-014, while significantly decreasing the Lachnospiraceae_NK4A136_group and Clostridium_sensu_stricto_1 abundance compared to LC diet. Rikenellaceae_RC9_gut_group is involved in carbohydrate degradation [43]. Eubacterium_coprostanoligenes can convert cholesterol into phytosterol, which helps reduce serum cholesterol and may produce short-chain fatty acids (SCFAs) to enhance intestinal health [44]. This might explain why the HC group had lower serum cholesterol than the LC group. Christensenellaceae_R-7 group is involved in the degradation of cellulose in the intestine [45]. However, in this study, the abundance of Christensenellaceae_R-7 group was negatively correlated with the cellulose content in the diet. Clostridia_UCG-014 is usually regarded as a pathogen, and its increased abundance is closely related to a variety of diseases [46,47,48]. Alistipes, a Gram-negative bacterium from the Bacteroides group, primarily produces succinic acid [49]. Treponema, the dominant bacteria in the sheep intestine, is closely related to the diet’s ratio of concentrate to roughage. Zhou et al. found [50] that treponema bacteria in the pig intestine are rich in a large number of polysaccharide-degrading enzyme genes. In addition, Treponema includes pathogenic bacteria [51,52], and an increase in its abundance under the conditions of an HC diet may have adverse effects on intestinal health. Ruminococcus, a typical anaerobic bacterium in the ruminant gastrointestinal tract, primarily participates in cellulose decomposition and fermentation [53] and it plays a key role in regulating the intestinal microecological balance [54]. Ruminococcus abundance increases in Crohn’s disease patients, and its metabolite glucorhamnan can induce dendritic cells to secrete the inflammatory cytokine TNF-α [55]. Lachnospiraceae_NK4A136 group can ferment indigestible carbohydrates, such as cellulose and hemicellulose, and it contributes to the maintenance of a healthy intestinal environment; its relative abundance is correlated with the ratio of dietary concentrate to coarseness [56,57]. The decrease in Lachnospiraceae_NK4A136 abundance may be related to the lower cellulose content in the HC group. This contradicts the findings of Chen et al. [12], who reported that an HC diet increased the abundance of Lachnospiraceae_NK4A136 group in the colon. The metabolites of Clostridium sensu stricto 1 are acetate, propionate, and butyrate [58], and some members of Clostridium sensu stricto 1 may participate in the regulation of the inflammatory response and help maintain the intestinal microecological balance [59]. In conclusion, these results demonstrate that the HC diet caused disorders of colon digestion and metabolism, and they explain the changes in colon fermentation parameters.

The metabolome results showed that the highly concentrated diet significantly increased the CA and CDCA concentrations. CDCA and CA are the main primary bile acids in humans, synthesized from cholesterol in hepatocytes. They undergo 7α-dehydroxylation by intestinal microorganisms to form secondary bile acids [60]. Both primary and secondary bile acids regulate host metabolism and immune responses [61]. Bacteroides [62], *Clostridium* perfringens [63], and *Ruminococcus* [64] are involved in converting primary bile acids into secondary bile acids. In this study, the increase in primary bile acid abundance in the HC group may be linked to the diet’s high starch content, leading to more concentrate entering the colon, consistent with previous findings [65]. Additionally, bile acid abundance was significantly positively correlated with *Ruminococcus*, *Alistipes*, *[Eubacterium]_coprostanoligenes_ group*, *Clostridia_UCG-014,* and Treponema, and it was significantly negatively correlated with *Lachnospiraceae_NK4A136_group*. Bile acids can reduce the triglyceride content in serum [66,67,68]. The decrease in triglycerides in the serum of the lambs in the HC group may be related to the increase in the bile acid concentration. This is consistent with the research of Yang et al. [69], who found that bile acid supplementation reduced serum triglyceride levels in chickens. In addition, bile acids function as inflammatory agents, stimulating the production of pro-inflammatory mediators through both Egr-1-dependent and -independent mechanisms [70]. Thus, bile acids play a key role in regulating inflammatory responses. In colitis patients, cholic acids inhibit peroxisome proliferator-activated receptor α, impairing fatty acid oxidation and intestinal stem cell renewal, which leads to increased epithelial damage [71]. Zheng et al. [72] found that the increase in bile acid caused by a short-term high-fat diet can aggravate colitis symptoms. lysoPA is an important inflammatory marker that directly induces cytokine and chemokine secretion and promotes inflammation [73,74]. In addition, lysoPA leads to impaired barrier function, and it has been found that tight junction protein expression is reduced in lysoPA-treated cells [75]. Therefore, the balance of intestinal lysoPA is essential for intestinal health. Methapyrilene is a hepatic toxin capable of causing increased levels of NADP^+^ and decreased levels of glutathione, causing oxidative stress [76]. Fusaric acid, a mycotoxin derived from the genus Fusarium, can cause apoptosis and increased inflammation [77], as well as inducing DNA damage [78] and lipid peroxidation [79]. Therefore, in this experiment, the damage caused by the high-concentrate diet to the colon may be related to the increase in the concentrations of the abovementioned metabolites.

It is well known that gastrointestinal microbes and metabolite alterations affect host metabolism and immunity [80,81,82]. Studies have found that an HC diet alters the microbial composition and structure in the gastrointestinal tract, leading to increased permeability and impaired barrier function, as well as gastrointestinal epithelial inflammation [12,82]. GPX4 and GSS are involved in glutathione metabolism. GPX4 can oxidize GSH to oxidized glutathione, thereby reducing peroxides to their corresponding alcohols and mitigating oxidative damage [83]. TRX contains redox-active disulfide bonds, which regulate the reduction/oxidation balance by scavenging active oxygen and play a vital role in the antioxidant system [84,85]. We found that the HC diet significantly inhibited the expression of GPX4, GSS, and TXN, indicating that feeding an HC diet decreases the expression of antioxidant-related genes, which may lead to oxidative damage to the colon epithelium. CDX2 is crucial for maintaining the homeostasis of intestinal epithelial cells and is involved in their regeneration and differentiation. The expression of CDX2 is decreased in ulcerative colitis [86], and the absence of CDX2 in intestinal epithelial cells also leads to macrophage infiltration, which results in chronic inflammation [87]. TLR2 and CXCL10 participate in the Toll-like receptor signaling pathway. TLR2 can recognize a wide range of pathogens [88]. A previous study showed that TLR2 silencing significantly reduced inflammatory factor expression and oxidative stress in LPS-induced granulosa cells, while TLR2 overexpression promoted inflammation and oxidative stress [89]. CXCL10 is a pro-inflammatory cytokine with increased expression in the colonic epithelium of mice with colitis [90]. IL-1R exists in two forms, namely, IL-1R1 and IL-1R2, with IL-1R1 being the primary receptor, and IL-1R signaling directly induces chemokine expression and reactive oxygen species-generating genes, which have detrimental effects during epithelial injury-induced colitis [91]. The results showed an increased expression of TLR2, CXCL10, and IL-1R1 in the HC group, while CDX2 expression decreased. The above indicates that an HC diet leads to oxidative stress and inflammatory responses in the colonic epithelium.

## 5. Conclusions

In conclusion, we found that an HC diet induces cavitation and inflammatory cell infiltration in the colonic epithelium, promotes the expression of serum inflammatory factors, and reduces antioxidant capacity. The HC diet altered the colonic microbial community structure in lambs, leading to increased VFA levels and a decreased pH. In addition, the HC diet increased the levels of pro-inflammatory metabolites. The HC diet led to colonic epithelial inflammatory damage and a reduced antioxidant capacity by affecting cytokine–cytokine receptor interactions, glutathione metabolism, and peroxisome signaling pathways. In summary, the high-concentrate diet caused epithelial inflammation and oxidative damage by affecting the interaction between the microbial flora in the lamb colon and metabolites and the host epithelium, which eventually disrupted colon homeostasis and had a negative impact on sheep health.

## Data Availability

The datasets presented in this study can be found in online repositories. The name of the repository and accession number can be found below: NCBI: PRJNA1165956 and PRJNA1166455.

## Abbreviations

The following abbreviations are used in this manuscript:

VFAs	Volatile fatty acids
ASVs	Amplicon sequence variants
PCoA	Principal coordinate analysis
LEfSe	Linear discriminant analysis (LDA) coupled with effect size measurements
OPLS-DA	Orthogonal partial least squares discrimination analysis
VIP	Variable important in projection
KEGG	Kyoto encyclopedia of genes and genomes
DEGs	Differentially expressed genes

## Appendix A

### Appendix A.1

**Table A1:** Composition and nutritional status of the experimental diets (%).

Items	LC	HC
Ingredients (% of DM)		
Mixed hay	70.00	30.00
Corn	28.80	53.00
Soybean meal	0.00	14.9
Calcium phosphate dibasic	0.00	0.10
Salt	0.70	0.50
Premix1	0.50	0.50
Sodium bicarbonate	0.00	1.00
Total	100.00	100.00
Nutrient levels (%)		
Crude protein	10.70	14.40
Soluble carbohydrates	37.50	49.90
Neutral detergent fiber	36.90	23.30
Calcium	1.40	0.70
Phosphorus	0.30	0.30
Acid detergent fiber	27.90	15.00
Soluble carbohydrates/NDF ratio	1.02	2.14
Metabolizable energy (MJ/kg)	9.00	10.40

Provided per kg of premix: Fe 25 mg; Zn 35 mg; Cu 9 mg; Co 0.1 mg; I 0.9 mg; Se 0.25 mg; Mn 19.5 mg; nicotinic acid 60 mg; vitamin E 15 U; vitamin A 3000 U; vitamin D3 1000 U. Metabolizable energy is a calculated value, and the rest of the nutritional indicators are measured values.

### Appendix A.2

**Table A2:** PCR primer sequences of the target genes.

Genes	Primer sequence (5′-3′)	Product Size/bp	Tm/°C
Claudin-1	F: GTGGATGTCGTGCGTGTC R: TAGTCCCAGCAGGATGCC	127	58
Occludin	F: AGCAGCAGTGGTAACTTGG R: TCCCGTCGTGTAGTCTGTT	111	58
ZO-1	F: CGAGCAGACGCAGAAAA R: GGCAGAAGATTGTGGTTGA	123	55
β-actin	F: CATCGTCCACCGCAAAT R: GCCATGCCAATCTCATCTC	103	56
GAPDH	F: GGTCGGAGTGAACGGATTTG R: TGGCAACGATGTCCACTTTG	83	59
